# Microneedle System Coated with Hydrogels Containing Protoporphyrin IX for Potential Application in Pharmaceutical Technology

**DOI:** 10.3390/mps7050073

**Published:** 2024-09-13

**Authors:** Beata Czarczynska-Goslinska, Tomasz Goslinski, Agata Roszak, Anna Froelich, Piotr Szyk, Dariusz T. Mlynarczyk, Lukasz Sobotta, Irena Budnik, Oliwia Kordyl, Tomasz Osmałek

**Affiliations:** 1 Chair and Department of Pharmaceutical Technology, Poznan University of Medical Sciences, Rokietnicka 3, 60-806 Poznan, Poland; aroszak40@gmail.com (A.R.); tosmalek@ump.edu.pl (T.O.); 2 Chair and Department of Chemical Technology of Drugs, Poznan University of Medical Sciences, Rokietnicka 3, 60-806 Poznan, Poland; s84493@student.ump.edu.pl (P.S.); mlynarczykd@ump.edu.pl (D.T.M.); 3 3D Printing Division, Chair and Department of Pharmaceutical Technology, Poznan University of Medical Sciences, Rokietnicka 3, 60-806 Poznan, Poland; froelich@ump.edu.pl (A.F.); irena.budnik@gmail.com (I.B.); okordyl@ump.edu.pl (O.K.); 4 Doctoral School, Poznan University of Medical Sciences, Bukowska 70, 60-812 Poznan, Poland; 5 Chair and Department of Inorganic and Analytical Chemistry, Poznan University of Medical Sciences, Rokietnicka 3, 60-806 Poznan, Poland; lsobotta@ump.edu.pl

**Keywords:** Franz diffusion cells, microneedles, poloxamer, protoporphyrin IX disodium salt, stereolithography

## Abstract

The article aims to outline the potential of treating malignant skin cancer with microneedles covered with polymer layers containing a photosensitizer—protoporphyrin IX disodium salt (PPIX). The usefulness of stereolithography (SLA), which is a form of 3D-printing technology, for the preparation of a microneedle system with protoporphyrin IX was demonstrated. The SLA method allowed for pyramid-shaped microneedles to be printed that were covered with three different 0.1% PPIX hydrogels based on sodium alginate, xanthan, and poloxamer. Rheological tests and microscopic analysis of the hydrogels were performed. Microneedles coated with two layers of poloxamer-based hydrogel containing 0.1% PPIX were subjected to release tests in Franz diffusion cells. The release profile of PPIX initially increased and then remained relatively constant. The amount of substance released after a four-hour test in three Franz cells was 0.2569 ± 0.0683 mg/cm^2^. Moreover, the acute toxicity of this type of microneedle was assessed using the Microtox system. The obtained results show the usefulness of further development studies on microneedles as carriers of photosensitizing agents.

## 1. Introduction

Cancer, including melanoma skin cancer (MSC) and non-melanoma skin cancer (NMSC), is a leading cause of death worldwide. Traditional treatments such as chemotherapy, surgery, cryotherapy, and radiation have several drawbacks, including low specificity and short effects [1]. Therefore, novel approaches need to be developed. In this regard, photodynamic therapy (PDT) might seem promising for treating skin cancer and pre-cancerous conditions [2].

PDT constitutes an interesting and effective alternative or supplement to traditional therapies in the fight against skin cancers. It is mainly limited to topical use and seems safe due to negligible effects on underlying structures. Moreover, it is relatively cheap. The base of PDT is a photodynamic reaction. The photosensitizing substance, also referred to as the photosensitizer (PS), should be delivered to the pathologically changed tissue and subsequently irradiated with light of an appropriate wavelength (preferably visible) [3]. The wavelength, as well as the substance’s absorbance, should be in the range of 600–800 nm, as below this range, most of the energy is absorbed by heme, and above it, by water [4]. The excitation of PS with light leads to the initiation of the photodynamic reaction with the generation of reactive oxygen species (ROS) [5,6]. There is also a novel approach to utilizing nanoparticles, as some of them, especially those from lanthanide- or actinide-doped metals, reveal upconverting properties [6,7,8]. The formed radicals destroy cellular organelles, activating apoptotic or necrotic cell death mechanisms [3]. Ideally, this effect should be limited to cancerously changed tissues. This includes not only cancer cells, but also endothelial cells that form blood vessels and roots between cancer cells and supply them with nutrients. The ability of specific PS to induce vascular collapse is dependent on (but not only on) the accumulation of the compound in that tissue. This can be tailored by chemical modification or by designing a proper drug delivery system for PS [9,10,11,12]. The irradiation of PS leads to calcium flowing into endothelial cells, which induces their morphological changes. This activates platelet aggregation and vascular occlusion as the blood contacts wall collagen [9,13]. The damage increases vessel permeability, which leads to edema, and cells that undergo death release specific molecules that activate the immune system [9,14]. The precise mechanism that stands behind the activation of the immune system is yet to be discovered. Some of the PSs are associated with factors provoking the release of damage-associated molecular patterns, which include high-mobility group box 1, calreticulin, HSP70, ATP, and interferon-1. These trigger innate immune system cells to further release pro-inflammatory factors. The activation of the innate immune system is linked to and necessary for the induction of the adaptive response. Damage-associated molecular patterns play a role in the promotion of antigen-presenting cells, such as dendritic cells, to uptake tumor-associated antigens. It has been found that the cellular remains left after PDT have an increased ability to escape from dendritic cell endosomes, thereby increasing their ability to present antigens. Augmentation of this step has an impact on further adaptive immunological responses and recruitment of CD8+ T lymphocytes, which eliminates distant lesions of cancer and affects immunological memory [15].

The group of PSs includes natural ones and synthetic dyes, such as porphyrins, phthalocyanines, chlorins, bacteriochlorins, phenothiazinium dyes, rose bengal, hypericin, and curcumin. The first photosensitizer clinically applied for cancer treatment was hematoporphyrin derivative (HpD), which was used in a purified form as porfimer sodium (Photofrin) [1,16,17]. Another compound applied for topical purposes in PDT is 5-aminolevulinic acid (5-ALA), as well as its ester methyl aminolevulinate (MAL) [18]. After exogenous administration, 5-ALA, which is a prometabolite and, thus, a precursor of heme, undergoes biochemical activation to protoporphyrin IX (PPIX). PPIX accumulates in cancer cells due to the low activity of ferrochelatase and high expression of 5-ALA influx transporters [19]. The exposition of tissue to PPIX and visible light initiates a photodynamic reaction with the generation of ROS and the destruction of malignant changes [20]. It is worth noting that ALA can be quickly eliminated from the tissue after the treatment, limiting the problem of prolonged photosensitivity (less than 24 h) [1].

Photosensitizing dyes, such as protoporphyrin IX, are mostly used in traditional topical dosage forms, which face low transfer efficiency to the site of action. Microneedles are reported to be more capable of enhancing drug transport across the skin than other transdermal delivery methods [21]. The dimensions of microneedles allow for the stratum corneum barrier to be overcome without stimulating the dermal nerve ends. Microneedles create micropores in the skin, and drugs from the skin surface can permeate into the dermal microcirculation [22,23]. Local drug administration does not induce side effects or premature drug degradation [23]. Therefore, the possibility of using modern microneedle systems or hydrogels that could improve the effectiveness of PDT was considered [24,25,26,27,28]. Microneedle-based formulations have been applied so far in various studies in which hydrogel-coated microneedle arrays were coated with multiple biologically or pharmaceutically active substances [29,30,31,32,33]. There are the following categories of microneedles: solid microneedles for tissue pretreatment, degradable/dissolvable microneedles, coated microneedles with water-soluble pharmaceutical formulations, and hollow microneedles. Microneedles have been fabricated from various materials, including silicon, metals, polymers, polysaccharides, and ceramics [34,35]. Solid-coated microneedles can be used to pierce the superficial skin layer and deliver the drug they are covered with or enable the transdermal delivery applied later [21].

**Scheme 1:**
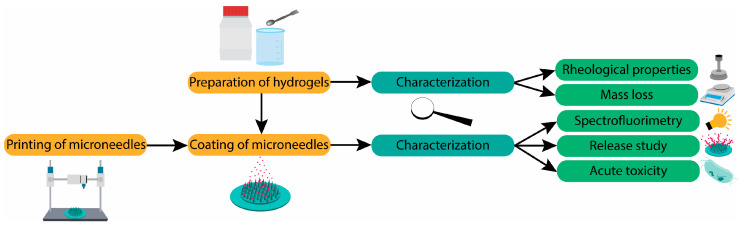
Summary of the methodology applied in the performed study.

Herein, we present the potential of a hydrogel-coated, solid microneedle system to achieve the topical delivery of PPIX disodium. We first evaluated various hydrogel formulations for microneedle coating and selected the best performing one for further studies. Then, we subjected the microneedles coated with 0.1% PPIX disodium salt poloxamer-based hydrogel to release studies in Franz diffusion cells for 4 h at seven time points. After the test, the amount of released substance was calculated. Finally, the acute toxicity of this type of microneedle was assessed using the Microtox system (Scheme 1).

## 2. Experimental Design

### 2.1. Materials

1. Sodium alginate (Sigma-Aldrich, St. Louis, MO, USA)
2. Poloxamer (Kolliphor^®^ P 407, Sigma-Aldrich, St. Louis, MO, USA)
3. Protoporphyrin disodium (Sigma-Aldrich, St. Louis, MO, USA)
4. Xanthan gum (POL-AURA, Dywity k/Olsztyna, Poland)
5. Ethyl alcohol 96% (POCH, Gliwice, Poland)
6. 2-Propanol (POCH, Gliwice, Poland)
7. Phosphate buffer pH 7,4 (concentrate, Chempur, Piekary Śląskie, Poland)
8. Light-curing resin (Phrozen Aqua Resin Blue, Hsinchu City, Taiwan)
9. Ultra-pure deionized water (HLP 10UV, Hydrolab, Straszyn, Poland)
10. Microtox Acute Reagent, Microtox Diluent, Microtox Osmotic Adjusting Solution, Microtox Reconstitution Solution (Modern Water plc, Cambridge, UK)

### 2.2. Equipment

- Magnetic stirrer with heating plate Heidolph MR Hei-Tec (Heidolph, Schwabach, Germany)
- Yellow line OST basic mixer (IKA, Staufen, Germany)
- Microscope Motic of B3 Professional Series (Motic, Xiamen, China) optical microscope equipped with Digital Moticam 2300 (Motic, Xiamen, China) camera
- Incubator WTB-BINDER KB 53/E2 (Binder + Co AG, Gleisdorf, Austria)
- Haake RheoStress 1 rheometer HAAKE^TM^ RheoStressTM1 (ThermoScientific, Waltham, MA, USA)
- Printer 3D Phrozen Sonic Mini 8k (Phrozen Tech Co., Ltd., Hsinchu, Taiwan)
- 3D Tronxy Moore 1 printer (Shenzhen Tronxy Technology Co., Ltd., Shenzhen, China)
- Anycubic Wash and Cure Machine v2.0 (Anycubic Technology Co., Ltd., Shenzhen, China)
- Portable WIFI digital microscope INSKAM316 (Shenzhen Yipincheng Technology Co., Ltd., Shenzhen, China)
- Spectrofluorimeter JASCO FP-6200 (Jasco, Tokyo, Japan)
- Franz diffusion cells (PermeGear, Hellertown, PA, USA)
- Tabletop SEM Microscope TM4000Plus Hitachi (Hitachi Ltd., Tokyo, Japan)
- Microtox M500 (Modern Water plc, Cambridge, UK)

## 3. Procedure

### 3.1. Preparation of Hydrogels

Use ultra-pure deionized water in all experiments. Prepare hydrogels by mixing the ingredients using a magnetic stirrer equipped with a heating plate or mechanical stirrer: the 1% sodium alginate-based hydrogel at 80 °C, the 1% xanthan-based hydrogel, and the 10% poloxamer-based hydrogel at room temperature (place the last one in the refrigerator overnight). For the preparation of the three respective 0.1% PPIX disodium salt hydrogels, follow the same procedure, but add PPIX disodium salt into hydrogel bases by introducing it into a mortar during stirring to obtain 0.1% concentration in hydrogels.

### 3.2. Optical Microscope Observation

Use a Microscope Motic B3-223 (Motic, Xiamen, China) to observe the distribution of PPIX disodium salt in the hydrogel bases. Use around 50 mg of each hydrogel and place it on separate microscope slides. Determine the shape, size, and dispersity of the hydrogel particles. Capture the images with a magnification of 40×.

### 3.3. Mass Loss on Drying

For the measurements of mass loss on drying, use the WTB-BINDER KB 53/E2 (Binder + Co AG, Gleisdorf, Austria) incubator. Weigh about 4.0 g of placebo hydrogels and 0.1% PPIX hydrogels on an analytical scale into plastic cosmetic jars to obtain three samples from each hydrogel. Place the samples in an incubator at 25 °C ± 0.5 °C for 6 h and weigh at the assumed time intervals: at time 0, after 15 min, 30 min, 45 min, 1 h, 2 h, 3 h, 4 h, 5 h, and 6 h. Calculate the mass loss of the sample over time and prepare charts illustrating the relationship between time and mass loss.

### 3.4. Rheological Properties of Hydrogels

Measure the rheological properties using a Haake RheoStress 1 rheometer (ThermoScientific, Waltham, MA, USA) equipped with parallel plate geometry (PP 35 Ti; measurement gap: 1 mm) and attached to the computer with RheoWin software (version 4.93; ThermoScientific, Waltham, MA, USA) at 25 °C. In the first step, set the zero point automatically, then move the upper plate upwards, place approximately 0.5 mL of the sample, and set the upper plate at 1 mm. Remove the excess amount of the sample from the lower plate. Perform constant rotation viscosity tests by varying the shear rate from 0.000 s^−1^ to 50.00 s^−1^ and from 50.00 s^−1^ to 0.000 s^−1^, with the cycle lasting 100 s.

### 3.5. Printing of Microneedles

**Figure 1:**
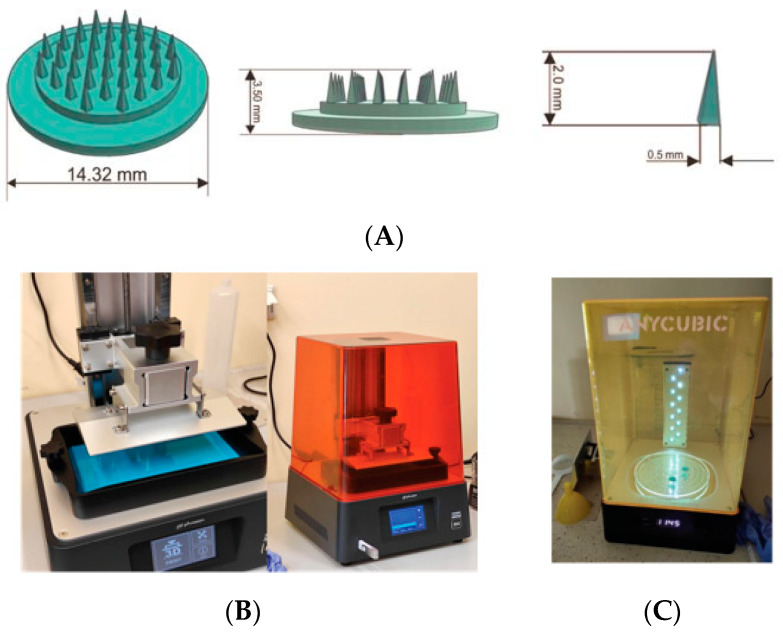
Technology applied for the preparation of microneedles: (**A**) Design of the microneedles; (**B**) Phrozen Sonic Mini 8k printer without and with the safety cover; (**C**) Anycubic Wash and Cure Machine.

Design a microneedle system consisting of pyramid-shaped needles located on a circular base with a diameter of 14.32 mm. The needle height is 2 mm, while the total height of a microneedle system is 3.5 mm (Figure 1A). Use CorelDRAW Graphics Suite 2023 and Tinkercad (https://www.tinkercad.com, accessed on 12 March 2023) to design the microneedle shape (Figure 1).

Prepare Phrozen Aqua-Blue photocurable resin. Use Printer 3D Phrozen Sonic Mini 8k (Phrozen Tech Co., Ltd., Hsinchu, Taiwan) (Figure 1B) and Anycubic Wash and Cure Machine v2.0 (Anycubic Technology Co., Ltd., Shenzhen, China) equipment for washing and drying of microneedles with stereolithography (Figure 1C). Before using the resin, shake it for one minute and then place it in a printer reservoir. Wait until the resin has completely degassed. Follow the printer guidelines and utilize an anti-UV cover. Separate the microneedle systems from the base plate using a scraper, put them in a container, and wash them with propan-2-ol.

In the next stage, subject the printed microneedles to curing on a rotating circular platform with UV light inside the Anycubic Wash and Cure Machine v2.0 (Anycubic Technology Co., Ltd., Shenzhen, China).

### 3.6. Coating of Microneedles

For coating of the microneedles, use a 3D Tronxy Moore 1 printer (Shenzhen Tronxy Technology Co., Ltd., No. 23 Baoya Road, Danzhutou Community, Longgang District, Shenzhen, Guangdong, China). To a Petri dish, add about 4 g of one of the prepared hydrogels with PPIX and place it on the device. Weigh each microneedle system, immerse the microneedles for coating, and take them out for drying and weighing. Wait 24 h and repeat the coating procedure.

Check the coating effects of microneedles with different hydrogels under the portable WIFI digital microscope INSKAM316 (Shenzhen Yipincheng Technology Co., Ltd., Shenzhen, China). Adjust the images manually, with magnification between 50 and 1000×.

### 3.7. Spectrofluorimetric Tests

Prepare the samples using an automatic pipette in 20 cm^3^ glass vials according to the following scheme:

(a) 3 vials—7.7 cm^3^ of ethanol;
(b) 3 vials—4.6 cm^3^ of previously prepared phosphate buffer (PB) + 3.1 cm^3^ of ethanol (ratio 60:40);
(c) 3 vials—7.7 cm^3^ of phosphate buffer;
(d) 3 vials—6.2 cm^3^ of phosphate buffer + 1.5 cm^3^ of ethanol (ratio 80:20).

After preparing solutions a-d, immerse the microneedles in the vials. In the case of 3 vials “a”, immerse separately microneedles coated with 0.1% PPIX alginate sodium-based, xanthan-based, or poloxamer-based hydrogel. Repeat the operation for the remaining vials, “b”, “c”, and “d”. Shake the samples gently, and then assess the release of PPIX by comparing the fluorescence of each sample using a spectrofluorimeter. Record the spectra of the samples in the emission mode in the wavelength range of 420–750 nm at an excitation wavelength of 400 nm. For sample “a”, use ethanol as a control; for sample “b”, use a buffer:ethanol mixture (60:40); for sample “c”, use phosphate buffer; and for sample “d”, use a mixture of buffer:ethanol (80:20).

### 3.8. The Release Study

Prepare the calibration curve for PPIX fluorescence in PB:ethanol 80:20 against different concentrations. Measure the fluorescence of the samples at the following concentrations: 0.0052, 0.0026, 0.0013, 0.00052, 0.00039, 0.00026, 0.000195, 0.00013, 0.000091, 0.000052, and 0.000026 mg/cm^3^ at the wavelength of 621 nm and triplicate the measurement. Calculate the slope of a linear function and use it in further studies to determine the concentration of the released PPIX.

For drug release experiments of microneedles coated with 0.1% PPIX hydrogel-based poloxamer, use Franz cells (PermeGear, Hellertown, PA, USA). Fill the three cells with 7.7 cm^3^ of acceptor solution composed of phosphate buffer (pH = 7.4) and ethanol at an 80:20 ratio (*v*/*v*). Prepare the buffer by mixing ready-made phosphate buffer concentrate (Chempur) and deionized water at a 1:25 ratio. Stir the acceptor fluid during the test at 200 rpm and keep it at a temperature of 32.0 ± 0.5 °C. Take the samples (1.0 cm^3^) from the receptor compartment using a syringe with a needle after 15 min, 35 min, 55 min, 75 min, 95 min, 120 min, and 240 min and replace them immediately with an equal volume of fresh receptor fluid. Replenish the resulting fluid loss in the cell with 1 cm^3^ of a pure buffer:ethanol mixture (80:20). For a fluorescence measurement, dilute 1 cm^3^ of the sample with a buffer:ethanol mixture (80:20) to 5 cm^3^ in the volumetric flask. Examine the collected samples using a spectrofluorimeter and determine the fluorescence at a wavelength of 621 nm.

Perform the tests for three microneedle systems (*n* = 3). Determine the PPIX concentration in the collected samples with the fluorimetric measurement at a wavelength of 621 nm. Perform the analysis of PPIX concentrations with a spectrofluorimeter JASCO FP-6200 (Jasco, Tokyo, Japan). Carry out the measurement in the emission mode at a wavelength ranging from 420 to 750 nm. Calculate the accumulated content of PPIX at selected time points according to the following Formula (1) [36]:

(1) $$Q=\frac{C_{n}\cdot V+\sum_{i=1}^{n-1}\left(C_{i}\cdot S\right)}{A}$$

where:

*Q*—the cumulative amount of PPIX;
*C_n_*—the concentration of active ingredient determined at the n^th^ sampling interval;
*V*—the volume of the Franz cell [cm^3^];
$\Sigma_{i=1}^{n-1}$—the sum of concentrations of active ingredient determined at sampling intervals 1 through *n* − 1;
*S*—the volume of the sample [cm^3^];
*A*—the diffusion surface [cm^2^], equal to 0.999 cm^2^, for the employed microneedles.

### 3.9. Acute Toxicity of the Coated Microneedles Measured Using Microtox Test

Perform the Microtox acute toxicity test according to a previously established procedure [37] using the Microtox M500 apparatus with Modern Water Microtox Omni 4.2 software. Modify the Microtox 81.9% screening test as follows: after measuring the bioluminescence of the *Aliivibrio fischeri* bacteria, precool 900 µL of Microtox Diluent (2% sodium chloride solution) to 15 °C, add to the suspension of *A. fischeri* and immediately immerse the microneedle disc fragment in the diluted bacterial suspension. Record changes in bioluminescence after 5 and 15 min upon the addition of the materials. Perform all experiments at least in duplicate.

## 4. Expected Results

### 4.1. Optical Microscope Visualization of Hydrogels

**Figure 2:**
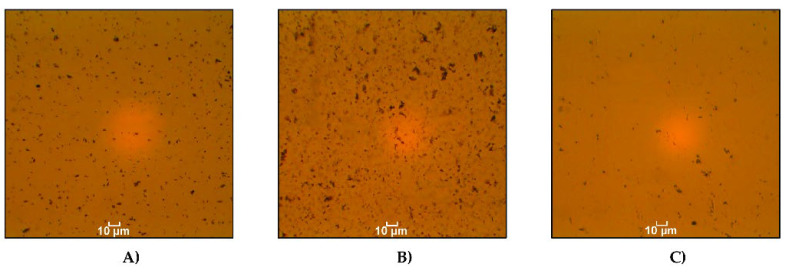
Microscopic image of 0.1% PPIX: (**A**) sodium alginate-based hydrogel, (**B**) xanthan-based hydrogel, and (**C**) poloxamer-based hydrogel at a magnification of 40×.

The prepared 0.1% PPIX hydrogels were studied under an optical microscope (Figure 2). In sodium alginate-based hydrogel (Figure 2A), dark PPIX aggregates of irregular shapes and irregular distributions were observed. The diameters of three randomly selected particles were 1.2 μm, 1.8 μm, and 1.8 μm. Moreover, subsequent aggregation of observed particles into larger particles was noted. In the xanthan-based hydrogel (Figure 2B), dark PPIX aggregates were observed. Their density was higher than that seen in the case of the sodium alginate-based hydrogel. The shapes were irregular, and their arrangement was chaotic. The diversity of the PPIX particle structures was higher than that previously seen in the alginate-based hydrogel. The diameters of randomly selected particles were 1.5 μm and 1.7 μm. Numerous aggregates with diameters of 5.2 μm, 3.6 μm, and 10.7 μm were also noted. In poloxamer-based hydrogel (Figure 2C), fine PPIX particles with dark color, irregular shapes, and irregular distribution were observed. The diameters of four randomly selected particles were 1.5 μm, 1.5 μm, 1.5 μm, and 1.8 μm. Moreover, a few aggregates were visible.

The obtained microparticles cannot enter the bloodstream directly because they are larger than 50 nm [38]. They should first dissolve under the skin to allow for further diffusion of the active substance. This is a beneficial effect from the point of view of the applications of these specific microneedles, which are intended to operate locally.

### 4.2. Mass Loss on Drying of Hydrogels

**Figure 3:**
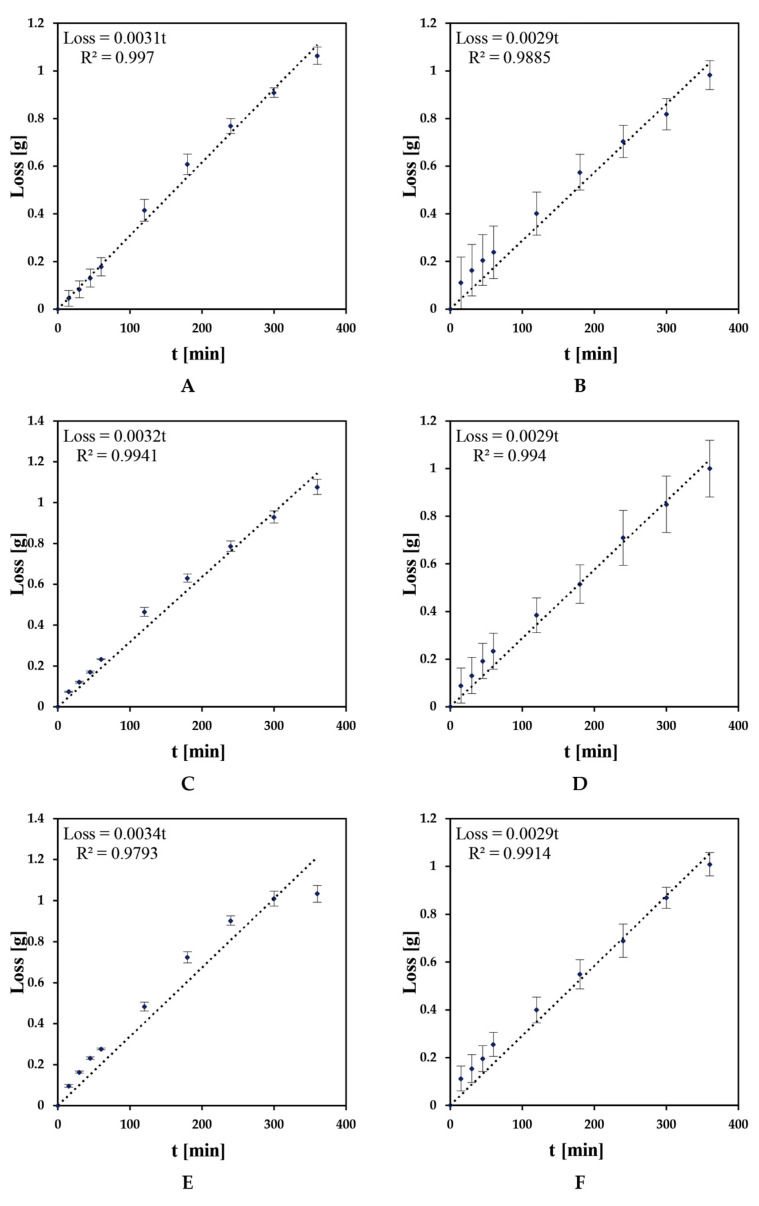
Mass loss of hydrogels over time for hydrogels based on sodium alginate (**A**) (placebo) and (**B**) (containing 0.1% PPIX); xanthan (**C**) (placebo) and (**D**) (containing 0.1% PPIX); and poloxamer (**E**) (placebo) and (**F**) (containing 0.1% PPIX). The error bars represent standard deviations from measurements performed in triplicate.

The mass loss on drying over time was performed in triplicate, calculated, and presented on the charts for all placebo hydrogels (Figure 3A,C,E) and the hydrogels containing PPIX (Figure 3B,D,F). For the sodium alginate-based placebo hydrogel, the initial mean weight was 4.1455 g, whereas for the xanthan-based and the poloxamer-based hydrogels, the initial mean weights were 4.5203 and 3.5851 g, respectively. The mass losses on drying of the tested types of placebo hydrogels were similar after 6 h and slightly exceeded 1 g in all cases. For the sodium alginate-based placebo hydrogel, the mean loss was 1.0640 g, whereas for the xanthan-based and the poloxamer-based hydrogels, the mean values of weight loss were 1.0763 and 1.0329 g, respectively. For the sodium alginate-based hydrogel with 0.1% PPIX, the initial mean weight was 4.8328 g, whereas for the xanthan-based and the poloxamer-based hydrogels, the initial mean weights were 5.37470 and 4.6268 g, respectively. The mass losses on drying of the tested types of hydrogels with 0.1% PPIX were similar after 6 h and were ca. 1 g in all cases. For the sodium alginate-based hydrogel with 0.1% PPIX, the mean loss was 0.9820 g, whereas for the xanthan-based hydrogel with 0.1% PPIX and the poloxamer-based hydrogel with 0.1% PPIX, the mean values of weight loss were 0.9997 and 1.0086 g, respectively.

In the case of the placebo hydrogels (Figure 3A,C) and hydrogels containing 0.1% PPIX (Figure 3B,D), linear mass loss over time was observed with R^2^ values from 0.9970 to 0.9941. The results of individual samples at specific time intervals were also comparable. In the case of the poloxamer-based placebo hydrogel (Figure 3E,F), a linear mass loss on drying over time could be observed, which slightly flattened out at the end of the test. The results of individual samples at specific time intervals were also comparable by 240 min. After that time, the mass loss on drying of the poloxamer-based placebo hydrogel did not change linearly over time by 360 min. The result was supported by lower R^2^ values of 0.9793 and 0.9914.

The drying curves presented in Figure 3 deviate slightly from the linear model, with a slightly higher evaporation rate at the initial stage. Comparing the outcomes of our experiments to the results described in the literature, this result is quite typical for polymer solutions or diluted gels which are used for microneedle coating. For example, Gu and Alexandridis presented a detailed study regarding the drying process of poloxamer 407 films, which can be compared to our research [39]. According to the authors, in the initial stages, the water content is higher, which corresponds to a higher evaporation rate. With the decrease in water content, the impact of hydrated polymer chains increases, and the evaporation rate also decreases. However, it must be emphasized that the samples analyzed in the literature study were different from the gels we applied in microneedle coating, mostly because of the much higher poloxamer concentration. In our study, only 10% of the polymer was used, which resulted in different drying curves and lower polymer impact. Similar drying behavior of alginate-bases samples was described by da Silva et al. [40]. However, the applied samples were also different from those investigated in our study. To the best of our knowledge, there are no comparable studies regarding the drying behavior of pure xanthan gum gels. It seems that the approximate linearity of the observed relationship between the sample mass and drying time was related to relatively low concentrations of the polymers we used. Regarding the practical implications of the sample behavior during drying, it must be emphasized that coating and drying rates are mostly important in technological terms. Extended evaporation time might be inconvenient in terms of the manufacturing process. However, in the prediction of factors potentially affecting the clinical efficacy of the analyzed systems, different tests should be considered, like the drug release rate.

### 4.3. Rheological Study of Gels

#### Placebo Hydrogels

**Figure 4:**
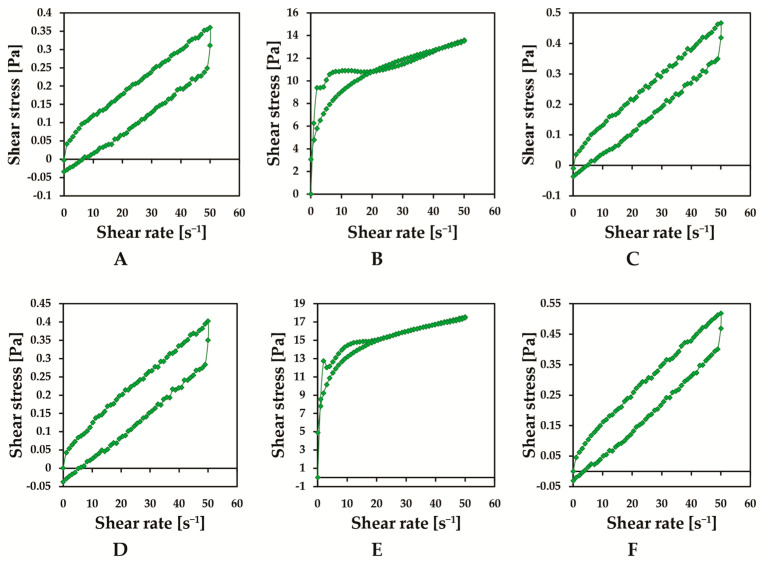
Plots of mean shear stress versus shear rate for different placebos ((**A**)—sodium alginate-based; (**B**)—xanthan-based; (**C**)—poloxamer-based) and 0.1% PPIX hydrogel samples ((**D**)—sodium alginate-based; (**E**)—xanthan-based; (**F**)—poloxamer-based).

The results of the rheological analyses, presented as stress vs. shear rate plots, are shown in Figure 4. The obtained curves were used to calculate the hysteresis loop area, and the ascending curve was fitted to the Herschel–Bulkley rheological model (Formula (2)) [41].

(2) $$\tau =\tau_{0}+K{\dot{\gamma }}^{n}$$

where τ is the shear stress, $\tau_{0}$ is the yield stress, *K* is the consistency index, $\dot{\gamma }$ is the shear rate, and *n* is the power law index. The obtained parameters are summarized in Table 1.

All investigated samples displayed non-Newtonian characters, as can be concluded from the observed values of the power law index. In all cases, *n* did not exceed 1, which is typical for shear-thinning systems, which exhibit decreased viscosity with the increase in the shear rate [41]. However, for both placebo and PPIX-loaded samples with sodium alginate and poloxamer, the *n* values were within the range of 0.85–0.90, while for xanthan gum-based gels, lower values were obtained. The observed phenomenon is related to a stronger viscosity drop in the experiment conducted for xanthan-based systems, while the behaviour of the samples with poloxamer and sodium alginate can be regarded as more similar to Newtonian fluid. Xanthan gum gels also revealed higher yield stress points and stronger thixotropic properties. It may be assumed that, in the case of xanthan-based samples, higher shear forces may be necessary to induce flow compared to the other systems. The observed properties may affect the process of microneedle dip coating. As already mentioned by Gill and Prausnitz [42], it can be expected that a more viscous system will form a thicker layer, which, in turn, may result in better drug-loading efficiency.

Regarding the shapes of the investigated samples, they were liquids of relatively low viscosity, except for the xanthan gum coating, which revealed higher viscosity. The visual inspection is basically consistent with the Herschel–Bulkley model parameters presented in Table 1. As was mentioned in Section 4.3, the *n* values for poloxamer- and alginate-based samples were closer to 1, while for the xanthan-based sample, it was lower, corresponding to more discernible shear-thinning properties. Moreover, the summarized yield stress points showed clear differences corresponding to the sample behavior. The values of yield stress and consistency index recorded for xanthan gum (placebo) revealed quite high variability. The possible explanation for this phenomenon may be related to problems with sample homogeneity, which might occur during mixing of the polymer with water. It must be emphasized that rheological equipment is quite sensitive and different results can be obtained when the analyzed sample reveals even small inhomogeneities which are not discernible in visual inspection. However, this problem occurred in the placebo sample only, and the further studies were primarily focused on poloxamer-coated microneedles. It is also noteworthy that no visible inhomogeneities were detected during sample preparation. Taking into consideration the area of hysteresis loops, it must be emphasized that, theoretically, thixotropy may mostly affect the coating process. It is vital in the case of multiple dipping leading to local disruption of the gel-forming forces, which, in the case of thixotropic formulations, need some specific recovery time. However, there are no data describing such a phenomenon in the literature. It seems that the thixotropic effects displayed by the studied samples were weak enough for this effect to be negligible, particularly in the case of poloxamer- and alginate-based gels.

### 4.4. The SLA Printing and Coating of the Microneedles Followed by SEM Assessment

**Figure 5:**
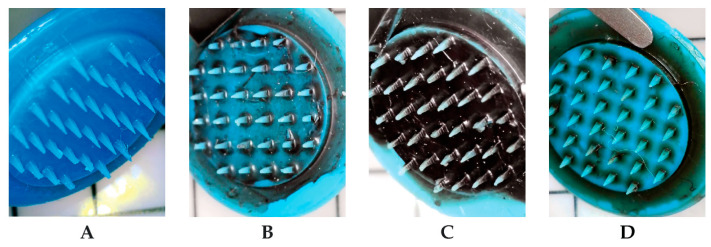
Photographs of microneedles: (**A**) uncoated; coated with 0.1% PPIX alginate sodium-based hydrogel (**B**); coated with 0.1% PPIX xanthan-based hydrogel (**C**); coated with 0.1% PPIX poloxamer-based hydrogel (**D**).

Microneedles were printed with SLA according to the designed drawing (Figure 5A). The same immersion depth in the coating material was used for all prints. After the first coating, the microneedles were left to dry until the next day. Then, the coating process with a second layer of the solution was performed for the second time. The microneedles were observed under the WIFI digital microscope INSKAM316.

**Figure 6:**
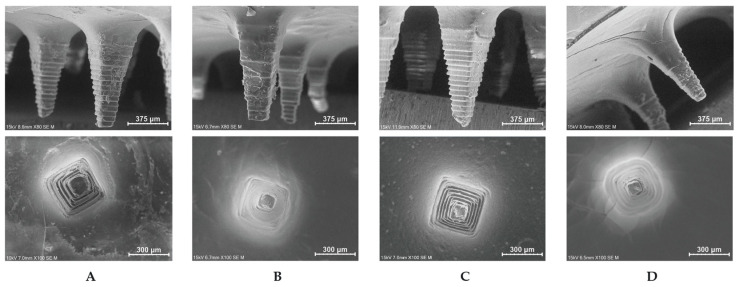
SEM images of microneedles from side (magnification 80) and above (magnification 100) of (**A**) uncoated hydrogel; (**B**) hydrogel coated with 0.1% PPIX alginate sodium-based; (**C**) hydrogel coated with 0.1% PPIX xanthan-based; and (**D**) 0.1% PPIX poloxamer-based hydrogel.

On each microneedle after the second coating (Figure 5B–D), the amount of hydrogel on the surface increased. This was confirmed by the mass difference calculation and both optical microscope and SEM assessment (Figure 6). The coating of the microneedles with hydrogels was not uniform over the entire print surface. This undesired effect can result from difficulties in refining the operation of the coating platform and from the physical properties (density) of hydrogels. The consistency and surface tension of the hydrogel may impact the quality of the coating layer, limiting its adhesion to the surface. This was observed for the coating of microneedles with 0.1% PPIX sodium alginate-based hydrogel (Figure 5B). This conclusion was also confirmed by the calculated mass differences, which, in the case of alginate, were insignificant. The difference in the weight after coating with the second polymer layer increased by an average of 0.3% compared to the weight of the microneedles without coating. Better coating results were observed when the 0.1% PPIX xanthan-based hydrogel was used (Figure 5C). In this case, the calculated average mass difference was 1.2%, which proved that there was better adhesion of the xanthan hydrogel with 0.1% PPIX than that of the sodium alginate hydrogel with 0.1% PPIX. The difference in the weight after coating with the second polymer layer increased by an average of 1.2% for the poloxamer-based 0.1% PPIX hydrogel, which proved that the adhesion of the poloxamer hydrogel was similar to that noted for the xanthan-based 0.1% PPIX hydrogel and much better than that for the sodium alginate-based 0.1% PPIX hydrogel.

Unfortunately, the drug formulation was coated on the backing layer of the microneedle and only on some parts of the microneedle shafts; the tips of the microneedles were uncoated. This phenomenon could have an adverse effect on the release of substances from microneedles and make the determination of the effective drug loading of the microneedle array impossible [42].

In the literature, the coated microneedle system was chosen to improve the penetrability of the pro-metabolite of 5-aminolevulinic acid (ALA) and to deliver it to a required depth of 2 to 3 mm from the surface of the skin [43]. Such an application would enable the delivery of ALA by creating micropores in the skin and making the treated place accessible for light irradiation. Painful procedures such as debulking of nodular lesions could be avoided [27]. Placing the photosensitizer in the polymer matrix during the manufacturing procedure is not possible because of the curing stage. The technological process involving UV irradiation could lead to the bleaching of the PPIX embedded in the polymer. Such issues were raised by Zhang et al. [44]. Therefore, in the presented study, the surface of the microneedle system was covered with the hydrogel containing PPIX. It is worth noting that coated microneedles possess better mechanical strength, which contributes to their passing through the skin barrier without breaking or bending [10].

As far as pain issues are concerned, pain is weaker during the application of solid microneedles than those of hypodermic needles, which involve fluid injection for drug delivery. This was proven in the study of Gill et al. [45], who investigated the pain for a hypodermic needle and microneedles with a range of lengths (480, 700, 960, and 1450 μm) and widths (160, 245 and 465 μm). The microneedles turned out to cause significantly less pain than a 26-gage hypodermic needle with an insertion depth of 5 mm. Gupta et al. [46] used a Visual Analog Scale (VAS) to compare pain acceptance associated with the application of hollow microneedles and hypodermic needles. However, the pain level reported during 4 mm subcutaneous microneedle insertion and hypodermic needle insertion turned out to be comparable. Therefore, solid microneedles seem to be a less painful drug delivery system than hollow microneedles and hypodermic needles. These conclusions were confirmed by the studies of Haq and co-workers [47]. They used mean VAS scores, verbal descriptions, and questionnaire responses from the participants to compare the pain levels caused by microneedles and the hypodermic needle. Again, the microneedles seemed significantly less painful and discomforting.

### 4.5. Preliminary Spectrofluorimetric Tests and Release Study of PPIX from Microneedle Systems

Spectrofluorimetric tests to determine the effectiveness of the release of PPIX substances from microneedles were carried out following literature reports. In a study conducted by Rosetti et al., the model was used due to the fact that protoporphyrin IX emits fluorescence after excitation with light of an appropriate wavelength [48]. Interestingly, the emission spectra presented here differed depending on the solvent used. The spectrum recorded in the ethanol presented mainly one sharp band, whereas in the PBS, the intensity dropped four times and showed two emission bands with additional band bathochromic shifting. Such a situation may be associated with proton or electron transfer between the excited PPIX and water. This probably results from water (PBS)–chromophore (PPIX) hydrogen bond disruption [49]. There are other hypotheses about the phenomenon of fluorescence quenching by water, i.e., it follows an energy gap law or resonance electronic-to-vibrational energy transfer via dipolar coupling [50]. On the other hand, the explanation of splitting bands and reducing the emitted signal in PBS may originate from aggregates of PPIX. The aggregation phenomenon of macrocyclic compounds is well known and occurs often [51]. Herein, mixtures of PBS and ethanol were used as solvents, and in these cases, lowering of signal intensities was not observed. Thus, it may be concluded that the cause of fluorescence quenching is an aggregation of PPIX, which was reduced by the addition of a co-solubilizer—ethanol.

**Figure 7:**
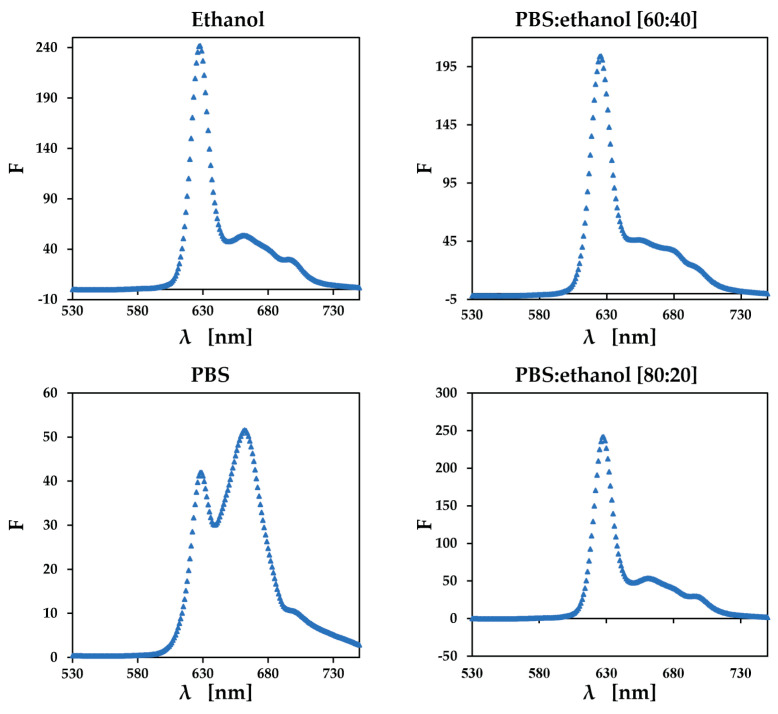
Fluorescence (F) of protoporphyrin 0.1% PPIX hydrogels in various solvents (Ethanol, PBS:ethanol [60:40], PBS, PBS:ethanol [80:20]), observed when examining microneedles coated with poloxamer-based gels. PBS—phosphate buffer pH 7.4.

The release of protoporphyrin 0.1% PPIX from the microneedles coated with sodium alginate-based, xanthan-based, and poloxamer-based hydrogels was assessed by fluorescence intensity (F). The effect was evaluated in various media, including ethanol, phosphate buffer with a pH of 7.4 (PBS), and ethanol:PBS combinations in proportions of 60:40 and 80:20. The highest values of recorded fluorescence were obtained in samples containing microneedles coated with a poloxamer-based hydrogel with PPIX (Figure 7). Additionally, the highest fluorescence intensity was noted when a buffer:ethanol mixture (80:20) was used as a solution for 0.1% PPIX. Based on the conclusions drawn, further tests were performed on microneedle samples coated with a poloxamer-based hydrogel in a buffer:ethanol mixture solution (80:20).

**Figure 8:**
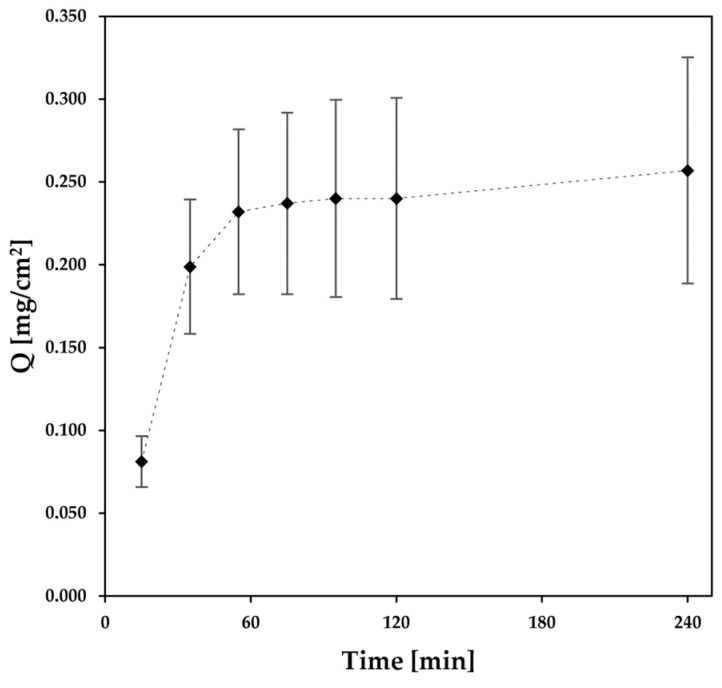
Graph of the amount of PPIX released over time from microneedles coated with poloxamer-based 0.1% PPIX hydrogel.

The release study was carried out in parallel in three Franz cells. As previously assumed, a buffer:ethanol mixture (80:20) was used as the release solution. Samples were taken after the following times: 15 min, 35 min, 55 min, 75 min, 95 min, 120 min, and 240 min. The fluorescence of collected samples was examined using a spectrofluorimeter at 621 nm (Table 2). The dependence of the average maximum fluorescence value was assessed against the concentration of PPIX (Figure 8). For the standard curve, the fluorescence assessment at 621 nm against each concentration was performed in triplicate.

The obtained concentrations were used to calculate the amount of substance released. Based on the results, the relationship between the amount of released substance and the time was plotted (Figure 8).

As can be observed in Figure 8, the amount of substance released initially increased over time by 60 min and then remained relatively constant by 240 min, which indicates that PPIX was released gradually from the microneedles throughout the study. The differences in the determined concentrations between the three cells resulted from the fact that the coating layer of the microneedle systems was non-uniform due to the coating process not yet being optimized. By adding up the amounts of the substance released at specific time points, the release per cm^2^ could be determined. Therefore, the cumulative average amount of the substance released from the three cells was 0.2569 ± 0.0683 mg/cm^2^.

Comparing the presented release profile to the results described in the literature, it can be noted that the variability obtained for the analyzed samples is usually quite high in the initial studies performed at the very beginning of the pharmaceutical development processes. Jadach et al. investigated the release profile of prototype microneedles made of the Phrozen Aqua Blue photocurable resin. However, their Lubrizol-based hydrogel coating contained clotrimazole (CLO). The cumulative CLO content per unit area turned out to demonstrate some variability as well [30]. In another study, Li et al. investigated microneedles coated with dyes and nanoparticles contained in a polyvinylpyrrolidone-based formulation. In the case of the PVP-K30 coating and three samples tested at each time point, the error bars were, again, relatively high. However, it must be emphasized that the reported release profile was performed in the skin in vitro [52].

### 4.6. Acute Toxicity of the Materials Measured Using Microtox Test

**Figure 9:**
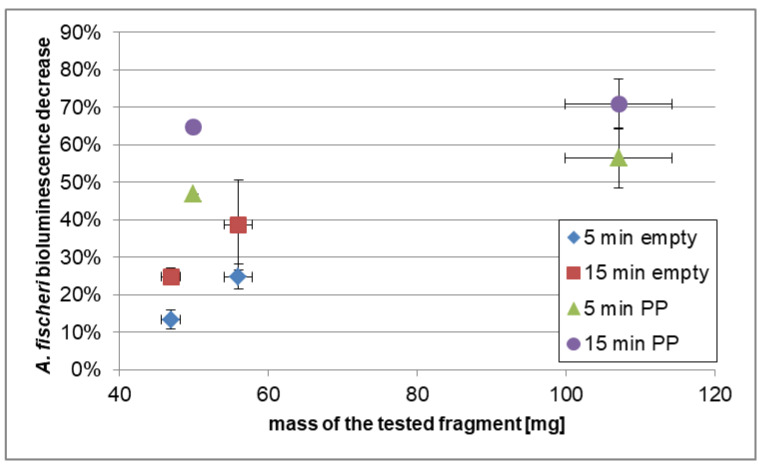
Changes in *Aliivibrio fischeri* bioluminescence upon contact with the uncovered pyramid-shaped microneedles (empty) and the pyramid-shaped microneedles covered with 0.1% PPIX disodium salt (PP) after 5 and 15 min.

The produced microneedles were assessed for their acute toxicity towards *Aliivibrio fischeri* bacteria in the Microtox test. The test is based on the change in the bacterial bioluminescence, as *A. fischeri*’s bioluminescence is connected with its metabolism, and thus, in the presence of a toxic substance, the bioluminescence decreases [53]. All the developed materials were subjected to the test. The results are summarized in Figure 9.

As can be seen by comparing the data obtained for each individual material, the addition of PPIX has a strong effect on the toxicity—in each case, the decrease in bioluminescence is higher for samples containing PPIX than for the samples without it. Another interesting finding is that the bioluminescence decreased as the contact time with the microneedles increased—the toxicity was more significant for the samples that were incubated for 15 min than for the samples with shorter incubation times. This phenomenon was observed regardless of the presence of the PPIX, which might have been released from the microneedles and was probably due to a component forming the microneedles, which might have been gradually leaching an aquatic toxicant to which *A. fischeri* are prone to. However, one should approach the results with caution, as due to the use of large chunks of the systems in the test, the calculated concentrations were well above the toxic concentration of polyacrylates forming the microneedles (EC_50_ of polymethacrylate 1000 mg/L) [54].

The type of resin and its biocompatibility are very important in PDT, where the microneedle system serves as a scaffold for the proper formulation. Three-dimensional printing is a fast, cheap method that allows for easy adaptation of the polymer scaffold to the type, shape, surface, and depth of the cancer lesion. It seems that polymer-based material is superior to other materials for such applications [30,55,56,57]. The applied resin is based on acrylates and is considered to have low toxicity and to be suitable for medical applications [58], as materials of this type are widely used in stomatology for dental prostheses [59]. As the producer claims, the Phrozen Resin Aqua Blue is engineered to facilitate 3D printing while delivering exceptional precision and producing robust, durable models that resist breakage. This resin achieves high-quality prints with detail and accuracy, ensuring minimal warping and exceptional dimensional stability to preserve the integrity of printed models. Technical specifications include a viscosity range of 75–175 cPs, a density of 1.12 g/cm^3^, and a surface hardness of 86 Shore D. The resin has a tensile strength (UTS) of 24 MPa, a tensile modulus of 588 MPa, an elongation at break (EAB) of 21%, and an Izod notched impact strength of 1.81 KJ/m^2^. After the proper post-processing, the printed microneedles are stable and do not degrade under normal conditions. This balanced performance makes Phrozen Resin Aqua in Blue a reliable choice for printing microneedles with proper mechanical properties [60].

## 5. Conclusions

The presented study indicates the usefulness of stereolithography for the preparation of a microneedle system with protoporphyrin IX. Pyramid-shaped microneedles were covered with three different 0.1% PPIX hydrogels based on sodium alginate, xanthan, and poloxamer. Mass loss on drying, rheological tests, and microscope analyses, including optical and SEM assessments, of the hydrogels were performed.

It is worth noting that rheological tests revealed shear-thinning properties in all of the investigated systems. It was shown that xanthan gum-based gels had stronger thixotropic properties and higher yield point values compared to alginate- and poloxamer-based samples. The studies have proven that all tested hydrogels have virtually no resistance to flow under the influence of the applied shear stress, as evidenced by the observed low value of dynamic viscosity. However, the resistance recorded in the case of xanthan-based hydrogels turned out to be higher than the resistance of other hydrogels. The tests showed no visible deviations between the rheological properties of placebo hydrogels and hydrogels with 0.1% PPIX, which proves that PPIX in the applied amount does not affect these properties.

Spectrofluorimetric studies have shown that the poloxamer-based PPIX hydrogel releases the active substance from the microneedles most effectively when a phosphate buffer:ethanol mixture (80:20 *v*/*v*) is used as a solvent. This conclusion was reached by also analyzing phosphate buffer:ethanol (60:20 *v*/*v*), ethanol, or phosphate buffer mixtures as elution fluids. The results recorded for the remaining hydrogels indicated significantly lower PPIX release. In the case of the xanthan-based hydrogel and the alginate-based hydrogel, significant results regarding released PPIX were obtained only when a phosphate buffer:ethanol mixture (60:40 *v*/*v*) was used as the elution fluid. Microneedles with two layers of poloxamer gel containing 0.1% PPIX were subjected to release tests in Franz diffusion cells within 4 h at seven time points. It was found that the release of PPIX initially increased and then remained relatively constant. The amount of substance released after a four-hour test in three cells was 0.2569 ± 0.0683 mg/cm^2^. Finally, the acute toxicity of this type of microneedle was assessed using the Microtox system. It turned out that the addition of 0.1% PPIX disodium salt had a noticeable effect on the toxicity of the studied microneedle system. In each case, a higher level of acute toxicity was observed for samples containing microneedle material covered with PPIX than for the uncovered ones.

The 3D printing technology seems to be an effective way of producing microneedles. However, the surface smoothness of these systems requires further optimization to eliminate the incompletely covered zones on the surfaces of microneedles.

## Figures and Tables

**Table 1:** Rheological parameters obtained for placebo hydrogel samples and PPIX-loaded hydrogel samples.

	Sample	Area of the Hysteresis Loop [Pa/s]	τ_0_ [Pa]	K [Pa·s*^n^*]	*n* [-]	r [-]
Placebo	Sodium alginate	5.18 ± 0.07	0.0405 ± 0.0048	0.0092 ± 0.0014	0.9065 ± 0.0112	0.9980 ± 0.0004
	Xanthan gum	23.11 ± 4.34	5.8827 ± 2.5215	2.3690 ± 1.9035	0.3263 ± 0.1222	0.9085 ± 0.0239
	Poloxamer	5.02 ± 0.21	0.0297 ± 0,0032	0.0123 ± 0.0018	0.9124 ± 0.0295	0.9990 0.0004
PPIX-loaded	Sodium alginate	5.14 ± 0.07	0.0304 ± 0.0033	0.0128 ± 0.0013	0.8590 ± 0.0262	0.9988 ± 0.0002
	Xanthan gum	24.58 ± 9.98	11.6800 ± 0.2263	0.8672 ± 0.0837	0.4831 ± 0.0204	0.9842 ± 0.0012
	Poloxamer	5.65 ± 0.54	0.0342 ± 0.0089	0.0170 ± 0.0032	0.8607 ± 0.0578	0.9987 ± 0.0004

**Table 2:** Fluorescence values and calculated concentrations at 621 nm at specified time intervals.

	Cell I		Cell II		Cell III	
t [min]	F	C (mg/cm^3^)	F	C (mg/cm^3^)	F	C (mg/cm^3^)
15	18.4489	0.01285	13.3928	0.00935	13.5683	0.00945
35	24.8898	0.01735	16.7500	0.01170	18.1072	0.01265
55	20.8312	0.01455	13.9585	0.00975	15.4101	0.01075
75	21.1131	0.01470	14.0391	0.00980	13.2224	0.00920
95	16.8990	0.01180	9.43245	0.00660	12.7960	0.00890
120	15.9796	0.01115	9.50608	0.00665	11.4026	0.00795
240	17.9541	0.01250	12.5054	0.00870	8.31079	0.00580

## Data Availability

Data will be made available on request.
